# Retracted: A Rare Case of Chronic Appendicitis Superimposed on an Incarcerated de Garengeot Hernia Prospectively Identified on Computed Tomography

**DOI:** 10.1155/2018/3183747

**Published:** 2018-10-23

**Authors:** Case Reports in Surgery

At the request of the authors, the article titled “A Rare Case of Chronic Appendicitis Superimposed on an Incarcerated de Garengeot Hernia Prospectively Identified on Computed Tomography” [1] has been retracted. There was disagreement between the authors in regards to the pathology results and final diagnosis, as chronic appendicitis could not be determined on the pathology. There is a De Garengeot hernia, however, chronic appendicitis is not a pathologic diagnosis and therefore there are no pathologic criteria to diagnose such an entity.
